# Characterization of calcifications in posterior horn of human meniscus using micro-computed tomography

**DOI:** 10.1016/j.ocarto.2026.100820

**Published:** 2026-05-16

**Authors:** Ville-Pauli Karjalainen, Iida Hellberg, Aleksandra Turkiewicz, Bijay Shakya, Nodira Khoshimova, Eeva Nevanranta, Shuvashis Das Gupta, Khaled Elkhouly, Amanda Sjögren, Mikko A.J. Finnilä, Patrik Önnerfjord, Velocity Hughes, Jon Tjörnstrand, Martin Englund, Simo Saarakkala

**Affiliations:** a Research Unit of Health Sciences and Technology, Faculty of Medicine, University of Oulu, Oulu, Finland; b Lund University, Faculty of Medicine, Department of Clinical Sciences Lund, Orthopaedics, Clinical Epidemiology Unit, Lund, Sweden; c Department of Biomedical Engineering, Lund University, Lund, Sweden; d Biocenter Oulu, University of Oulu, Oulu, Finland; e Lund University, Faculty of Medicine, Department of Clinical Sciences Lund, Rheumatology and Molecular Skeletal Biology, Lund, Sweden; f Skane University Hospital, Department of Clinical Sciences Lund, Orthopaedics, Lund University, Lund, Sweden; g Department of Diagnostic Radiology, Oulu University Hospital, Oulu, Finland

**Keywords:** Meniscus, Calcification, Osteoarthritis, Micro-computed tomography, Basic calcium phosphate, Calcium pyrophosphate

## Abstract

**Objective:**

Meniscal calcifications are associated with meniscal degeneration and osteoarthritis (OA). We investigated micro-computed tomography (μCT) imaging for identification of calcification patterns caused by basic calcium phosphate (BCP) and calcium pyrophosphate (CPP).

**Design:**

Posterior horns of human medial and lateral menisci from 19 individuals with medial compartment knee OA and 21 deceased donors were imaged with high-resolution μCT. Raman spectroscopy characterized the calcification types from histological sections, adjacent to μCT piece. Qualitative and quantitative analysis, visualization, and grading were performed using 3D μCT images of meniscal calcifications.

**Results:**

Different calcification patterns were observed with BCP and CPP. BCP was found at the borders of meniscal tissue and inside complex tears or fibrillation. In contrast, CPP accumulated as circumferential rod-like structures between collagen bundles, mainly inside the meniscal tissue, and lacked the porous 3D structure observed in BCP calcifications. Quantitatively, CPP samples had a higher calcification volume compared to BCP with a geometric mean ratio of 14 (95%CI; 3,73), and larger particle sizes with a ratio of 7 (95%CI; 2,8). Additionally, BCP calcifications had an organized porous structure with a closed porosity range of 6–19%, while a similar structure was not seen in CPPs (range 1–4.5%).

**Conclusions:**

We qualitatively and quantitatively identified volumetric and morphological differences in the calcification deposition patterns between BCP and CPP calcifications in human meniscus. Ultimately, this μCT imaging method supports more detailed characterization of calcifications in OA and helps to pave the way for identifying potential biomarkers and understanding the mechanistic models of disease progression related to pathological calcifications better.

## 1 Introduction

Calcifications in the meniscus are suggested to be an essential part of meniscal degeneration and osteoarthritis (OA) [[1], [2], [3]]. Meniscal calcifications propose a predisposing factor for cartilage lesions and OA progression, and therefore, this ectopic mineralization should be taken into consideration when developing OA treatments [4,5]. Currently, there are no effective disease-modifying drugs for OA, but some drugs have shown potential in inhibiting the pathological calcification [6,7]. Understanding the different calcification deposition patterns caused by different calcification types may increase the knowledge regarding calcification processes in OA and other diseases with crystal deposition involvement, which could, consequently, help in future drug development.

Calcium pyrophosphate (CPP) and basic calcium phosphate (BCP), such as hydroxyapatite are the most common types of calcifications found in the knee joint, including meniscus, synovial fluid, and articular cartilage [8,9]. BCP calcifications have been associated with OA and inflammation, while CPP is related to chondrocalcinosis and aging [7,8,10,11]. Methods such as Raman spectroscopy and scanning electron microscopy with energy dispersive analysis (SEM-ED) can differentiate BCP and CPP calcifications, but they can only analyze small samples. Furthermore, imaging with multi-energy computed tomography (MECT) combined with photon-counting detectors (PCDs) has previously enabled the differentiation of calcifications in 3D with varying accuracy [7,12].

We have previously shown that micro-computed tomography (μCT) can visualize and quantitatively analyze meniscal soft tissues and calcifications in 3D [5,[13], [14], [15], [16]]. High-resolution μCT imaging enables non-destructive assessment of relatively large samples, provides accurate 3D information of the entire structure, and detects minute calcified particles that are often missed by conventional 2D techniques. We therefore propose that combining high-resolution μCT with Raman spectroscopy would enable identification of different calcification patterns within different calcification types. Since the posterior horn has shown to be the most susceptible region to degeneration and tearing in the meniscus, we focused on this region in the current study [17].

This study aimed to utilize high-resolution μCT to quantitatively characterize and study the calcifications in human menisci *ex vivo*. The objectives were to volumetrically characterize and compare the deposition patterns of BCP and CPP crystal types in the medial and lateral meniscus posterior horns of osteoarthritic knees from total knee replacement (TKR) patients and donors without clinical knee OA.

## 2 Method

### 2.1 Tissue samples

This study was approved by the regional ethical review board at Lund University (Dnr 2015/39, Dnr 2016/865, and Dnr 2019/00323). The sample set of this study consisted of 80 meniscus samples obtained from the knee tissue biobank MENIX, located at Skåne University Hospital in Lund, Sweden. We selected both the medial and lateral menisci from a single knee from 19 individuals with end-stage medial compartment knee OA, who underwent a TKR and 21 deceased adult donors without a known diagnosis of knee OA. More details on tissue samples can be found in Supplementary Material 1.

### 2.2 Sample preparation

After receiving the meniscus samples from the MENIX biobank, they were thawed in phosphate-buffered saline and fixed in 4% saline-buffered formaldehyde. Supplementary Material Figs. S1 and S2 show images of thawed donor and TKR menisci before any sample processing. In macroscopic images, the largest CPP calcifications can be seen by eye, circulating inside the meniscus, aligning with circumferential collagen fiber bundles (Fig. S1). No cases of visible calcifications in the BCP samples were seen. More details on sample preparation in Supplementary Material 2.

### 2.3 Raman micro-spectroscopy

The Raman spectroscopy measurements and results are explained in more detail in our previous study that used partly the same sample set as this work (all 74 samples in this study were also used in the previous work) [8]. More details on Raman spectroscopy can also be found in Supplementary Material 3. BCP deposits generate an intense sharp peak at 960 cm^−1^ and CPP at 1049 cm^−1^ in the Raman spectrum, which can be used to characterize the calcification type [8,18]. The samples were divided into following groups based on the Raman results; BCP, CPP, unidentified (if no calcifications were present in the Raman sections), or both (if both BCP and CPP spectra were found in the same sample).

### 2.4 Micro-computed tomography imaging

Adjacent to the histological sections, fixed μCT samples were dehydrated with ethanol, treated with hexamethyldisilazane (HMDS) and imaged with a desktop μCT device (SkyScan 1272, Bruker MicroCT). Image acquisition and details can be found in Supplementary Material 4. Six out of 80 samples were omitted from further analysis due to image acquisition failure with sample rotation motor with the μCT device, resulting in a total of 74 samples.

### 2.5 Grading of meniscal samples

The calcifications in the menisci were graded between 0 and 5 from no calcifications to widespread calcifications from the 3D μCT images according to Hellberg et al. [5]. Three individuals (VPK, IH, NK) graded the samples individually and then attained a consensus grade that was used as the final grade.

The histopathological scoring and corresponding results are explained in more detail in our previous study that used the partially same sample set as in this work [8]. Briefly, the histopathological scoring of meniscal degeneration was conducted as described by Pauli et al. [1]. Two independent graders (VPK & IH) graded the samples individually and then similarly attained a consensus grade that was used as the final grade.

### 2.6 Micro-computed tomography analysis of meniscal calcifications

The whole μCT pieces were used for the analyzes. A sharpening filter followed by thresholding, and despeckling were applied using CTAn software (Version 1.20.2 Bruker microCT). We calculated the total calcification volume and meniscus tissue volume using the full 3D image stacks. In addition, for each sample, we performed individual object analysis, which describes the 3D morphology of each individual calcification particle within each sample. It gives the following parameters as an output: number of particles, individual calcification particle volume, surface to volume ratio, sphericity, and closed porosity volume. Surface to volume ratio describes the complexity or roughness of calcification surface with higher value indicating more complexity. High sphericity indicates a sphere-like morphology, while lower values indicate rod-like or other more complex shapes.

### 2.7 Statistical analysis

SPSS (IBM SPSS Statistics, v. 29.0, NY, USA) was used for statistical analysis. For all parameters, we did a group-wise comparison between BCP and CPP groups using linear mixed models. All parameters were transformed into logarithms before calculating arithmetic mean for each meniscus which was used as outcome in the statistical models. The results were back transformed and thus, the difference between groups are expressed as ratios of geometric means. More details on statistical analysis in Supplementary Material 5.

## 3 Results

### 3.1 Descriptive statistics on the study participants

The study subjects had an average age of 70.6 years, and average height of 170 cm with half of them being females. (Table 1). BCP calcifications were present in total 9 females and 8 males in at least one meniscus, while CPP calcifications were present in 1 female and 4 males in at least one meniscus.

**Table 1:** Descriptive statistics on all study subjects, divided by OA status, and then divided by calcification types.

Group	No. Female/male	Age (years) mean (SD)	Knee left/right	Height (cm) mean (SD)	BMI mean (SD)
All study subjects	20/20	70.6 (6.6)	21/19	169.8 (10.9)	26.5 (4.5)
**By OA status**					
OA	10/9	69.8.0 (6.4)	13/6	170.4 (10.3)	30.5 (4.6)
Donors	10/11	71.4 (6.8)	8/13	169.3 (11.4)	23.4 (4.4)
**By calcification group**^a^					
Unidentified	13/11	70.3 (6.9)	12/12	168.4 (11.2)	24.3 (4.2)
BCP	9/8	76.0 (6.4)	12/6	176.0 (10.3)	28.5 (4.2)
CPP	1/4	78.0 (6.8)	3/2	165.0 (8.3)	25.3 (3.9)
Both	1/1	78.0 (n/a)	1/1	160.0 (n/a)	31.4 (n/a)

a One donor can be included in more than one group, if medial and lateral menisci had different calcification types.

Raman measurements identified BCP in 28, CPP in 7, and both calcification types in two samples within the 74 meniscus samples that were imaged with μCT device. All BCP calcifications were found in OA menisci, while CPP particles were found equally often in OA menisci and donors. More details about the Raman results can be found in our previous study [8]. Furthermore, in 37 samples, we found minute calcification like particles in the 3D μCT images and volumetric analyses, but since they were not detected with Raman microspectroscopy, this group is called unidentified.

### 3.2 Qualitative assessment of meniscal calcification patterns from micro-computed tomography imaging and histology

**Fig. 1:**
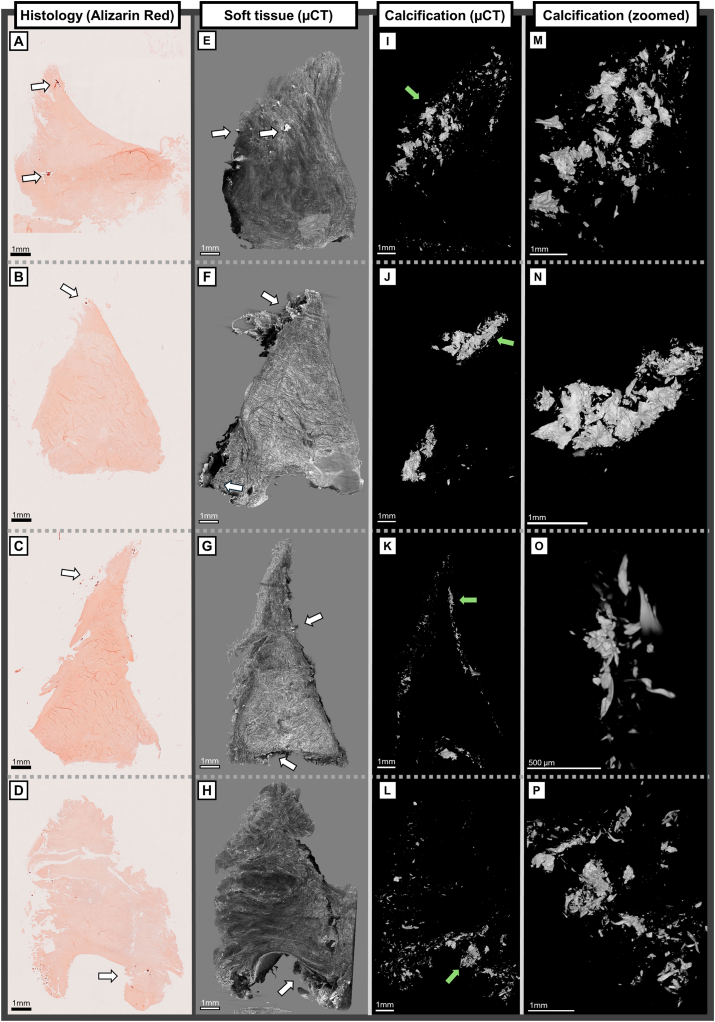
A, B, C, D) Overview of Alizarin red stained histological sections with BCP calcifications from different meniscus samples from individuals with OA. White arrows show areas with positive staining marking meniscal calcifications. The samples had Pauli scores of A = 16, B = 16, C = 15, and D = 17, respectively. E, F, G, H) 3D μCT images from meniscus soft tissue reconstruction show calcifications in white and meniscus tissue in gray. While some BCP particles seem to be inside the soft tissue in histology, from 3D μCT images it can be observed that they are located in the periphery of the meniscus, stuck inside complex 3D tears, or near fibrillations. Arrows highlight the calcifications in the image. I, J, K, L) 3D μCT images from meniscus calcification reconstruction. BCPs appear as punctate aggregates, measured 5–100 μm in largest diameter, but can also accumulate into larger clusters of calcifications, sized even between 0.5 mm and 1 mm. The green arrows highlight the area of the zoomed image. M, N, O, P) The zoomed calcification images show sharp morphology of the BCP calcifications, a likely contributor of tearing and degradation that advance the state of fibrillation while aiding the calcifications to bury in the surface fibrillations.

Visual examination of the μCT images revealed different calcification patterns between BCP and CPP samples. In Fig. 1, Fig. 3 3D μCT images show how BCPs are spread mostly on the surface, periphery and near the tears of meniscus. While few BCP particles seem to be inside the soft tissue in both 3D μCT and histology, they are in fact located inside complex tears or fibrillations of the meniscus. Similarly, Supplementary Video 1 shows BCP calcifications in an OA meniscus, mainly located on the surfaces and inside fibrillations. They appear mostly aggregated as punctate and small in size (5–100 μm in diameter), while larger clusters can be over 500 μm in the largest diameter and have characteristically sharp and pointy edges. Compared to the histology, not only larger volumes of calcifications are seen, but more diminutive calcifications can also be identified in 3D μCT.

Supplementary video related to this article can be found at https://doi.org/10.1016/j.ocarto.2026.100820

The following are the supplementary data related to this article.

Multimedia component 3

Multimedia component 4

Multimedia component 5

Multimedia component 6

**Fig. 2:**
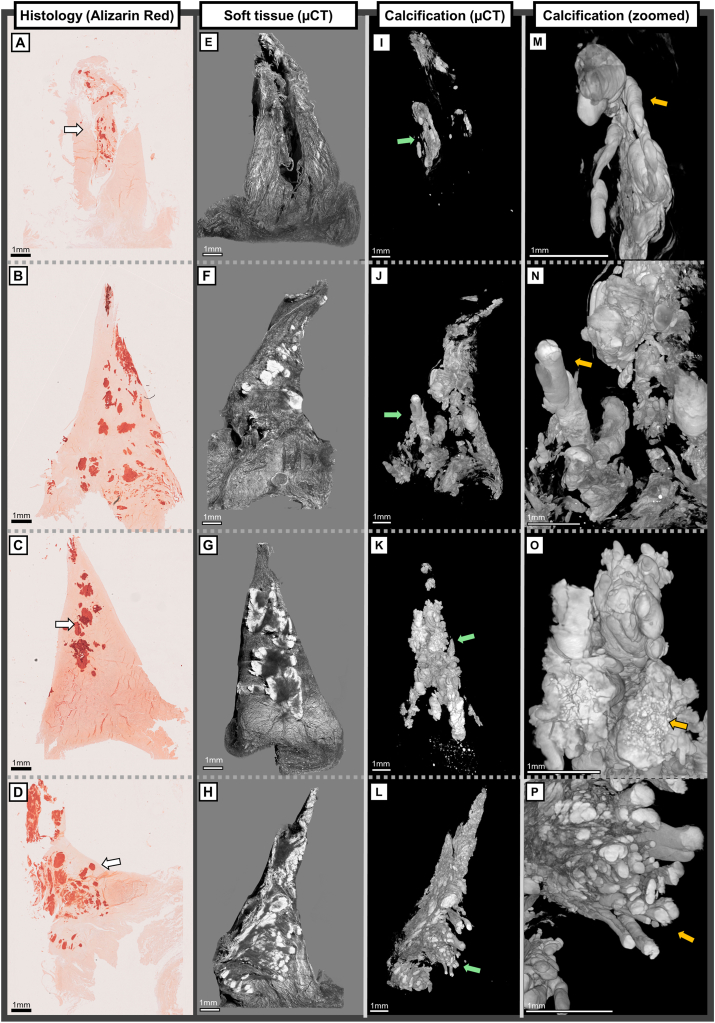
A, B, C, D) Overview of Alizarin red stained histological sections with calcium pyrophosphate (CPP) calcifications from four different deceased donor menisci. 2D sections show mostly spherical calcifications inside the tissue with more amorphous structures on the periphery and surface. The samples had Pauli scores of A = 14, B = 13, C = 13, and D = 14, respectively. E, F, G, H) 3D μCT images from meniscus soft tissue reconstructions show calcifications in white and meniscus tissue in gray. Similar to 2D histological sections, the calcifications cover large areas inside the meniscus. I, J, K, L) 3D μCT images from meniscus calcification reconstructions. As shown in 3D, long, rod-shaped calcification formations cover large areas inside the meniscus, while less dense, amorphous calcified structures are located on the surface and between the more solid calcifications. Green arrows show the zoomed area of calcifications. M, N) Smooth, rod-shaped calcifications align with the circumferential collagen fiber bundles inside the meniscus (orange arrow). O) Smooth aggregates of calcifications inside the tissue are accompanied by more amorphous calcification structures that are located on the tibial surface or between the solid calcifications (orange arrow). P) Multiple rod-shaped calcifications cover almost the whole meniscus (orange arrow).

CPP deposits appear mostly inside the meniscus tissue as long rod-shaped formations or ellipsoid-shaped clusters oriented along the circumferential collagen fibers (Fig. 2). Supplementary videos 2&3 show meniscus samples with CPP calcifications covering most of the tissue. We observed that the cross-sectional diameter of these solid rods was commonly between 0.2 and 1 mm. The largest calcification clusters seemingly accumulate between the circumferential collagen fiber bundles, aligning their largest diameter with the fibers, while pushing and forcing the collagen to warp around them. The CPP rods can cover most of the meniscus from anterior horn to posterior horn, as seen in Supplementary Figure S1 where thick CPP calcification can be seen inside the meniscus through the surface macroscopically. Furthermore, few cases of long and hollow calcifications were observed (Supplementary Figure S3, Supplementary Video 3). Even smaller, ellipsoid-shaped calcifications align with the circumferential collagen orientation. However, when seen on the surface of meniscus, CPP calcifications are spread along the surface instead of along the circumferential collagen network. Additionally, CPP calcifications have generally smooth surfaces when appearing inside the tissue. Amorphous, less dense structures of CPP are seen near the cylinder-shaped calcifications and on the surface of the meniscus. Differences between solid rod-shaped and more amorphous CPP calcifications can be seen in Fig. 2I, K.

**Fig. 3:**
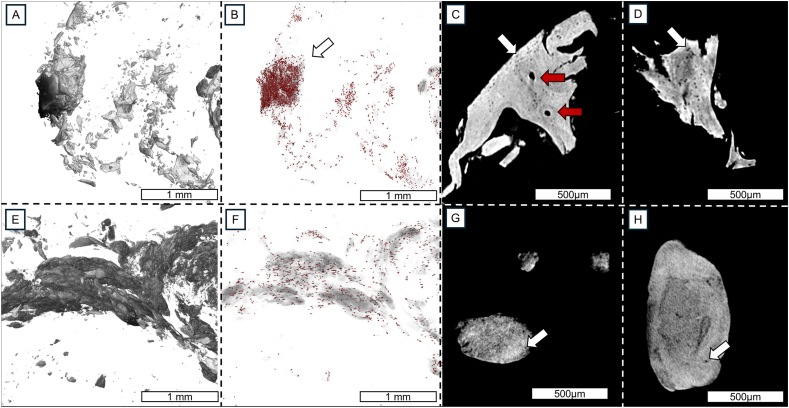
Closed porous structures inside the calcifications. A) 3D μCT image of a BCP cluster located in the outer periphery of a meniscus from an individual with OA. The sample had a Pauli score of 16. B) Lacunae-like pores inside the calcifications are depicted in red color. C) 2D cross-section of a BCP calcification cluster from Fig. 3B, shows organized lacunae-like pores inside the BCP calcification (white arrows). Red arrows depict continuous, open channels–resembling Haversian canals in cortical bone–going through the calcification cluster. D) A similar 2D cross-section of a BCP cluster from a different sample shows the same porous and organized structure inside the BCP calcifications. E) CPP calcifications from the same sample as A). F) Closed porous structures appear in lower density than in BCP calcifications. G) 2D cross-section of a CPP calcification shows some porous structures inside the calcification, but to a lesser extent than in BCP calcifications (white arrow). H) 2D cross-section of a CPP cluster from a different sample shows a similar structure with only a few lacunae-like pores (white arrow).

Qualitative description of calcifications, cells, and proteoglycan content in the meniscus from histopathological sections are seen in Supplementary Material 6. Two example µCT images of meniscus samples, one with both BCP and CPP calcifications and other with only BCP calcifications (as identified with Raman), together with images of Alizarin red, H&E, and Safranin O–Fast Green stained sections are shown in Supplementary Figs. S4 and S5. Examples of Raman spectra for both BCP and CPP can be found in Supplementary Fig. S6.

### 3.3 Porosity of calcifications

μCT inspection of the BCP calcifications revealed a porous, organized 3D structure (Fig. 3A–D) that was consistently observed across all samples with BCP calcifications. While some pores were observed inside the CPP calcifications in μCT images, their amount was substantially lower than in BCP calcifications (Fig. 3E–H). Supplementary Video 4 of a close-up BCP calcification with segmented pores shows how constant the pore structure is. Supplementary Figure S4 shows Alizarin red, H&E, and Safranin O-Fast Green stained sections of the same sample. Few pores or fractures with cell lacunae are seen inside the CPP calcifications, but no pores or cell lacunae inside the BCP particles are seen from the histological sections.

### 3.4 Grading of meniscal calcifications

**Fig. 4:**
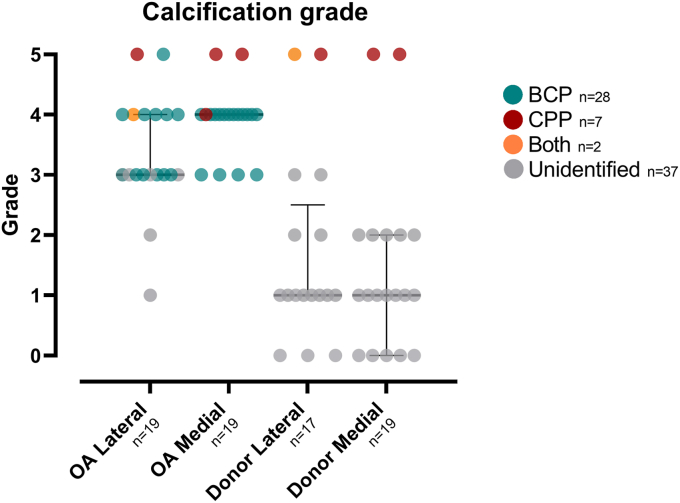
The relationship between OA status and joint compartment with calcification grade. The calcification grades of individual menisci within the four sample groups are shown together with medians and their interquartile ranges. The calcification types are highlighted with different colors. The calcification grades were higher in individuals with OA compared to deceased donors. In addition, the menisci with CPP calcifications had higher calcification grades than samples with BCP calcifications. Only eight donor samples had a grade 0, meaning that there were no visible calcifications in them.

The calcifications were graded in each meniscus sample from the 3D μCT images. As visualized in Fig. 4, the median calcification grade was higher in individuals with OA compared to deceased donors [median (1st quartile, 3rd quartile)]: OA Lateral [3 (3,4)], OA Medial [4 (4,4)], Donor Lateral [1 (1,2.5)], Donor Medial [1 (0,2)]. The inter-observer reliability was assessed between readers and is found in Supplementary material 7. Furthermore, even though donor menisci had lower median calcification grades compared to OA menisci, high grades were observed also in this group in samples with CPP calcifications. Eight donor samples had a grade 0, meaning that there were no visually detectable calcifications in them.

**Fig. 5:**
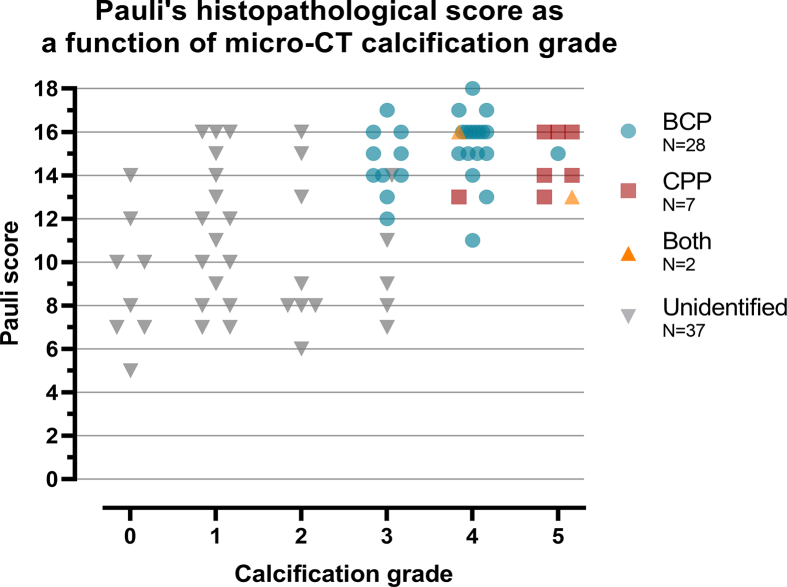
The relationship between the histopathological degeneration (Pauli score) and the micro-computed tomography (μCT) calcification grade.

The calcification grade increased in with the increasing severity of histopathological degeneration (Pauli score) (Fig. 5). In meniscus samples showing moderate to severe degeneration (Pauli score ≥10), two distinct calcification patterns emerged: one characterized by small, minute deposits (grades 1–2), and another by larger to more extensive calcifications (grades 3–5).

The histopathological results are explained in more detail in our previous study that used partly the same dataset as in this work [8]. Briefly, the Pauli's histopathological scoring showed higher scores for medial and lateral OA menisci, when compared to medial and lateral donor menisci. Moreover, BCP was more associated with histopathological degeneration compared to CPP.

### 3.5 Quantitative 3D calcification analysis from micro-computed tomography images

**Fig. 6:**
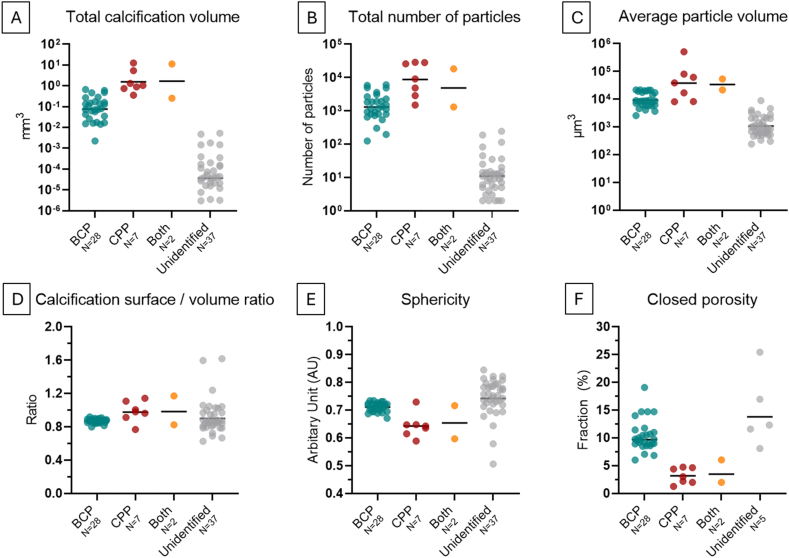
Quantitative 3D analysis of calcifications A) Geometric means of total calcification volume in each sample. B) Geometric means of total number of calcification particles. C) Geometric means of average particle volumes. D) Geometric mean calcification surface/volume ratio. E) Geometric mean sphericity. F) Geometric mean closed porosity. For closed porosity, only samples that had particles large enough to have closed porosity were included in the unidentified group.

BCP and CPP calcifications were quantitatively analyzed in 3D from the μCT images (Fig. 6, Table 2). Based on the linear mixed models, we found that the total calcification volume was higher in CPP compared to BCP (Fig. 6A). Similarly, the total number of particles was greater in CPP than in BCP (Fig. 6B). The average particle volume was also larger in CPP (Fig. 6C). Furthermore, the surface-to-volume ratio was higher in CPP (Fig. 6D), while sphericity was greater in BCP (Fig. 6E). Lastly, porosity was higher in BCP compared to CPP (Fig. 6F). The total number of calcifications analyzed per meniscus is seen in Supplementary Fig. S7.

**Table 2:** Quantitative parameters for BCP, CPP, and their difference presented as geometric means.

Parameter	BCP (geometric mean)	CPP (geometric mean)	Ratio between CPP vs BCP (95% CI)
Total calcification volume (mm^3^)	0.075	1.014	14 (3–73)
Total number of particles	1178	8716	7 (3–17)
Average particle volume (μm^3^)	9419	37650	4 (2–8)
Surface to volume ratio	0.845	0.983	1.16 (1.08–1.25)
Sphericity	0.698	0.624	0.89 (0.83–0.95)
Closed porosity (%)	9.87	3.76	0.38 (0.23–0.63)

## 4 Discussion

This study investigated calcification patterns in human posterior horn menisci *ex vivo* using high-resolution 3D μCT imaging. Morphological and volumetric differences were identified between BCP and CPP deposits. BCP calcifications were typically located on meniscal surfaces and near tears, while CPP deposits were embedded within the tissue, aligned with circumferential collagen fibers. Quantitative analysis supported by statistical analysis revealed distinct differences between BCP and CPP calcifications, namely that BCP deposits had lower total volume, fewer and smaller particles, and lower surface-to-volume ratios compared to CPP, whereas BCP showed higher sphericity and closed porosity. These findings demonstrate that μCT imaging enables reliable differentiation between BCP and CPP calcifications in human meniscus.

Our findings suggest that BCP accumulation and morphology may play a direct role in meniscal degradation in OA. Qualitatively, BCP crystals were primarily located on the surface, periphery, and within complex meniscal tears. Given the calcifying potential of OA meniscal cells and the absence of intact surrounding tissue, some particles may have migrated through joint cavity from other joint structures, such as articular cartilage, where calcified crystals are also commonly found [9,19]. BCPs appeared in punctate patterns but formed sharp-edged clusters when aggregated. Previous studies have linked BCPs to destructive processes in conditions like Milwaukee shoulder syndrome and intraosseous migration, and intra-articular mineralization has been associated with knee pain [20,21]. Based on these studies and our results, we hypothesize that these sharp clusters may influence biomechanical properties of the tissue and contribute to mechanical degradation during joint movement.

In our samples, CPP calcifications were predominantly located within the meniscus, forming large rod-like structures aligned with the circumferential collagen bundles. In some cases, smaller, discontinuous clusters were observed in similar orientations, potentially representing precursors to the rod-like form. The smooth, elongated morphology of these calcifications suggests they occupy fluid channels between collagen fibers, consistent with known pathways where fluid flows during biomechanical loading [22,23]. Furthermore, CPPs were found on meniscal surfaces and tears as amorphous, randomly oriented deposits, distinct from the organized rods inside the meniscus. Surface-to-volume analysis revealed CPPs have more complex surfaces, while BCP particles were more spherical. Notably, BCPs exhibited a porous structure that was absent in CPPs, as confirmed by both qualitative and quantitative porosity assessments.

CPP calcifications in the meniscus may be associated with vascular structures, as seen in 3D μCT images showing complex, curved calcifications oriented nonlinearly toward tibial or femoral surfaces. Hollow, vessel-like calcified structures suggest vascular involvement. This aligns with prior findings linking arterial calcifications and atherosclerosis to OA [24], and calcification clusters have been reported to form near the largest vascular supply in the outer region of porcine meniscus [14]. The shape of these calcified hollow structures resembles meniscal vascularity that has been studied previously with μCT [14,25]. The shape of these structures resembles previously characterized meniscal vasculature but was observed only in CPP samples, while arterial calcifications have been reported to typically contain hydroxyapatite [26]. The absence of small particles inside these structures suggests that CPP likely forms locally from excess calcium ions that combine with pyrophosphate to form calcium pyrophosphate dihydrate crystals rather than being transported via the vasculature [27,28].

The closed porosity of BCP deposits consisted of organized porous structures inside the calcifications, which was not observed in CPP deposits to similar extent. In H&E-stained sections, no pore structures were visible inside BCP calcifications, while few were seen inside the fractures of larger CPP particles. However, in 3D μCT, the BCP had a highly porous structure, highlighting the benefit of our 3D method. Accurate differentiation of BCP and CPP calcifications in the soft tissues has been challenging with current imaging methods. Previous characterizing methods, like Raman and SEM-ED, require destructive sample processing, while current imaging methods, like MECT may not have sufficient resolution and accuracy [[29], [30], [31]]. The organized network of porous structures in BCP calcifications could therefore be used as an imaging biomarker for characterization of BCP from CPP calcifications with high-resolution imaging modalities like μCT and MECT combined with PCDs in future studies.

Based on the 3D μCT images and videos, we identified important differences between the BCP and CPP calcification patterns. As previously stated, BCPs were smaller, punctate, and found in the peripheral area, surface, and tears, spread along evenly with few larger clusters. While some amorphous CPP were on the surfaces of samples, the largest volumes of CPP were located inside the meniscus, along the circumferential collagen fibers, forming long rod-like formations. Similarly, a previous study has reported that in 2D, CPP formed round colonies that disrupt the collagen fiber organization [18]. Furthermore, BCPs were found both qualitatively and quantitatively in smaller volumes, fewer in number, and smaller in average particle size compared to CPP. Previously, these aggregates of BCP and CPP have been suggested to be approximately between 1 and 20 μm in diameter [32]. For comparison, our volumetric results for BCP and CPP were 9419 μm^3^ and 37650 μm^3^, which can produce approximated sphere diameters of 26 μm and 42 μm, respectively, which are higher than previously reported diameters from histological sections. The discrepancy between the previous studies and our results can be from our results including 3D information from larger samples.

Meniscal calcification may be linked to danger-associated molecular patterns in the knee joint, as microcrystals like BCP can trigger pro-inflammatory responses and promote chondrocyte hypertrophy [33,34]. We saw positive Safranin O staining, which marks an increase in proteoglycan content, and hypertrophic cells in the vicinity of BCP calcifications. Furthermore, BCP has been shown to induce hypertrophic differentiation [35,36], a key step in endochondral ossification, where cartilage transitions to bone through chondrocyte proliferation, hypertrophy, and apoptosis [37,38]. Apoptotic chondrocytes are associated with BCP crystals and may contribute to OA via calcifying apoptotic bodies [39,40]. Therefore, a similar fate of meniscal cells undergoing calcification may happen in the meniscus to hypertrophic chondrocyte-like cells that are near BCP deposits. It has been suggested that molecular mechanisms that control regular calcification in skeletal tissues are similar to calcification mechanisms in soft tissues [41]. Our μCT analysis revealed that BCP calcifications form porous, lacunae-like structures resembling osteocyte organization in subchondral bone and calcified cartilage. This pattern was consistently observed in 3D across all BCP samples but absent in CPP calcifications, indicating distinct calcification mechanisms. The unique architecture of BCP may be attributed to its high hydroxyapatite content, which plays a key role in bone regeneration [42]. These findings suggest that BCP accumulation in the meniscus mimics native bone tissue formation, supporting the hypothesis that soft tissue calcification may share molecular pathways with physiological skeletal mineralization.

We found only a few cases which had both calcification types in the same menisci, and in those cases, they were observed in different areas. In those samples, BCPs covered a relatively low volume compared to the whole calcification volume. Likewise, there has been incidents where both crystal types have been observed in articular cartilage [43]. CPP crystal formation is closely associated with the regulation of extracellular inorganic pyrophosphate, which also inhibits BCP crystal growth [44]. Dysregulation of pyrophosphate can be related to aging or genetic mutations, which leads to increased pyrophosphate crystal deposition [45]. CPP calcifications have been associated with senescent chondrocytes [35]. The cells around solid, rod-like CPP calcifications were elliptic-shaped, while cells around amorphous CPP calcifications seemed more hypertrophic. As the surface of solid CPP is smooth and arranged along the circumferential collagen fibers, this could minimize the disrupting effect the calcification cluster has on the meniscus function and account for the asymptomatic CPPD disease. Previous studies have shown increase in type X collagen near calcified fibrocartilage of meniscus, which acts as a facilitator and regulator for matrix mineralization [46,47]. This could suggest that after initial inflammation and formation of the calcification inside the meniscus, the smooth calcification particle does not severely disrupt the surrounding meniscal ECM.

Calcifications were present in all OA menisci, with BCP being the predominant type and correlated with Pauli's histopathology score [8]. Consistently, higher calcification grades were associated with increased Pauli scores in our previous study [5]. Notably, samples with CPP deposits exhibited the highest average calcification grades. A minimum grade of 3 was required for calcifications to be detectable in histological sections or via Raman spectroscopy, with grade 3 deposits approximating a total volume of 0.005 mm^3^ or 0.01% of total meniscus volume.

In the unidentified group, including 37 meniscus samples, very minute calcification particles were detected with μCT imaging. However, these small particles were not seen in any histological sections. As calcifications in the meniscus are not homogenously spread in the tissue, conventional 5 μm sectioning of the samples can easily miss these particles or they can tear off during the cutting process. As shown in volumetric results, the calcification volumes in the unidentified group are less than a fraction of meniscal total volume. This highlights the need for higher resolution analysis methods like μCT, as conventional histological sectioning is not necessarily sensitive enough for the smallest particles that are unevenly spread across the tissue. Although only 8 of 74 samples lacked visible calcifications on qualitative 3D μCT assessment, all samples contained minute particles, as shown with quantitative volumetric analysis, suggesting early microcrystal formation. These findings in OA-free menisci add to previous reports of widespread calcifications in OA meniscus [48], articular cartilage [9,43,49], and synovial fluid [50]. Given the average age of 71 and mild to moderate degeneration in our cohort, our results support the notion that minor calcifications are common with aging and probably BCPs [5,49]. Thus, future studies should aim at quantifying what volume of calcifications could be indicative of OA disease. Potential OA therapies could consider calcification inhibition as a potential disease-modifying strategy.

A limitation of the study was that only a portion of the meniscus posterior horn was imaged, which enabled optimal resolution due to μCT's geometrical magnification constraints. Despite the limited size, the imaged volume was substantially larger than in conventional 2D histology. As an additional limitation, since BCP calcifications were found in all OA tissue donors but one, and CPP only in few OA donors and deceased donors, we did not correlate their presence with any other potential donor-related clinical characteristics.

This study lays the groundwork on how μCT imaging can non-destructively reveal microstructural differences between BCP and CPP deposits in meniscal tissue. Although these features are beyond the resolution of clinical CT, defining their three-dimensional characteristics offers a useful reference point as clinical imaging technologies continue to advance. Our results further highlight that the two calcification types form in consistent and morphologically distinct ways, providing a foundation for future studies aimed at understanding the metabolic, mechanical, or inflammatory conditions that lead to different types of mineral deposition. Ultimately, this μCT imaging method supports more detailed characterization of calcifications in OA and helps to pave the way for identifying potential biomarkers and understanding the mechanistic models of disease progression related to pathological calcifications better.

## Author contributions

Conception and design: VPK, IH, SS, ME, AT. Provision of study materials and tissue preparation: ME, VH, PÖ, JT, VPK, KE, AS, SS. Micro-computed tomography imaging and analysis: VPK, EN. Raman spectroscopy and analysis: BS. Histological analysis: VPK, IH, NK. Statistical analysis: AT, VPK. Interpretation of results: All coauthors. Drafting of the article: VPK. Critical revision of the article for important intellectual content: All coauthors, Final approval of the article: All coauthors.

## Role of the funding source

The funders had no role in study design, data collection and analysis, decision to publish, or preparation of the manuscript.

## Data availability

The data underlying this article will be shared at a reasonable request to the corresponding author.

## Funding

This research has received financial support from the Research Council of Finland (grants no. 347445), Sigrid Juselius Foundation, Jane and Aatos Erkko Foundation, The Swedish Research Council, Österlund Foundation, Gustaf V 80-Year Birthday Foundation, Governmental Funding of Clinical Research within National Health Service (ALF), the Swedish Rheumatism Association, the Greta and Johan Kock Foundation, and the Foundation for People with Movement Disability in Skåne.

We would like to acknowledge the NORDFORSK grant from the project Molecular and structural biomarkers for personalized care in osteoarthritis (Project No.: 116406).

VPK has received funding from Finnish Cultural Foundation (Grant no. 00220451).

IH has received funding from Instrumentarium Science Foundation (Grant no. 210036).

## Conflict of interest

ME reports past consultancy for Grünenthal Sweden AB, Key2Compliance AB and Genascence.

Given their role as Associate Editor, Simo Saarakkala had no involvement in the peer-review of this article and has no access to information regarding its peer-review. Full responsibility for the editorial process for this article was delegated to another journal editor.

The other authors report no conflicts of interest.

## References

[1] Pauli C., Grogan S.P., Patil S., Otsuki S., Hasegawa A., Koziol J. (2011). Macroscopic and histopathologic analysis of human knee menisci in aging and osteoarthritis. Osteoarthr. Cartil..

[2] Sun Y., Mauerhan D.R., Honeycutt P.R., Kneisl J.S., Norton H.J., Zinchenko N. (2010). Calcium deposition in osteoarthritic meniscus and meniscal cell culture. Arthritis Res. Ther..

[3] MacMullan P.A., McCarthy G.M. (2010). The meniscus, calcification and osteoarthritis: a pathologic team. Arthritis Res. Ther..

[4] Sun Y., Mauerhan D.R. (2012). Meniscal calcification, pathogenesis and implications. Curr. Opin. Rheumatol..

[5] Hellberg I., Karjalainen V.P., Finnilä M.A.J., Jonsson E., Turkiewicz A., Önnerfjord P. (2023). 3D analysis and grading of calcifications from ex vivo human meniscus. Osteoarthr. Cartil..

[6] Nasi S., So A., Combes C., Daudon M., Busso N. (2016). Interleukin-6 and chondrocyte mineralisation act in tandem to promote experimental osteoarthritis. Ann. Rheum. Dis..

[7] Bernabei I., So A., Busso N., Nasi S. (2022). Cartilage calcification in osteoarthritis: mechanisms and clinical relevance. Nat. Rev. Rheumatol..

[8] Shakya B.R., Karjalainen V.P., Hellberg I., Finnilä M.A.J., Elkhouly K., Sjögren A. (2024). Prevalence and classification of meniscal calcifications in the human knee. Osteoarthr. Cartil..

[9] Stücker S., Koßlowski F., Buchholz A., Lohmann C.H., Bertrand J. (2024). High frequency of BCP, but less CPP crystal-mediated calcification in cartilage and synovial membrane of osteoarthritis patients. Osteoarthr. Cartil..

[10] Ea H.K., Chobaz V., Nguyen C., Nasi S., van Lent P., Daudon M. (2013). Pathogenic role of basic calcium phosphate crystals in destructive arthropathies. PLoS One.

[11] Fuerst M., Bertrand J., Lammers L., Dreier R., Echtermeyer F., Nitschke Y. (2009). Calcification of articular cartilage in human osteoarthritis. Arthritis Rheum..

[12] Stamp L.K., Anderson N.G., Becce F., Rajeswari M., Polson M., Guyen O. (2019). Clinical utility of multi-energy spectral photon-counting computed tomography in crystal arthritis. Arthritis Rheumatol..

[13] Karjalainen V.P., Kestilä I., Finnilä M.A., Folkesson E., Turkiewicz A., Önnerfjord P. (2021). Quantitative three-dimensional collagen orientation analysis of human meniscus posterior horn in health and osteoarthritis using micro-computed tomography. Osteoarthr. Cartil..

[14] Karjalainen V.P., Herrera Millar V.R., Silvia Modina |, Peretti G.M., Pallaoro M., Khaled Elkhouly | (2024). Age and anatomical region-related differences in vascularization of the porcine meniscus using microcomputed tomography imaging. J. Orthop. Res..

[15] Kestilä I., Folkesson E., Finnilä M.A., Turkiewicz A., Önnerfjord P., Hughes V. (2019). Three-dimensional microstructure of human meniscus posterior horn in health and osteoarthritis. Osteoarthr. Cartil..

[16] Sergio M., Karjalainen V.P., Das Gupta S., Herrera Millar V.R., Mirra G., Di Giancamillo M. (2025). Prolonged excessive weight induces spontaneous meniscal degeneration in sows: a preclinical model for obesity-related knee OA. Annals of Anatomy - Anatomischer Anzeiger..

[17] Englund M., Guermazi A., Gale D., Hunter D.J., Aliabadi P., Clancy M. (2008). Incidental meniscal findings on knee MRI in middle-aged and elderly persons. N. Engl. J. Med..

[18] Katsamenis O.L., Karoutsos V., Kontostanos K., Panagiotopoulos E.C., Papadaki H., Bouropoulos N. (2012). Microstructural characterization of CPPD and hydroxyapatite crystal depositions on human menisci. Cryst. Res. Technol..

[19] Kiraly A.J., Roberts A., Cox M., Mauerhan D., Hanley E., Sun Y. (2017). Comparison of meniscal cell-mediated and chondrocyte-mediated calcification. Open Orthop. J..

[20] Halverson P.B., Cheung H.S., Mccarty D.J., Garancis J., Mandel N. (1981). “Milwaukee shoulder”—association of microspheroids containing hydroxyapatite crystals, active collagenase, and neutral protease with rotator cuff defects. ii. synovial fluid studies. Arthritis Rheum..

[21] Collinot J.A., Pascart T., Budzik J.F., Hügle T., Hussenot M., Becce F. (2020). Non-invasive characterization of intra-articular mineralization using dual-energy computed tomography. Rheumatology.

[22] Agustoni G., Maritz J., Kennedy J., Bonomo F.P., Bordas S.P.A., Barrera O. (2021). High resolution micro-computed tomography reveals a network of collagen channels in the body Region of the knee meniscus. Ann. Biomed. Eng..

[23] Vetri V., Dragnevski K., Tkaczyk M., Zingales M., Marchiori G., Lopomo N.F. (2019). Advanced microscopy analysis of the micro-nanoscale architecture of human menisci. Sci. Rep..

[24] Macêdo M.B., Santos V.M.O.S., Pereira R.M.R., Fuller R. (2022). Association between osteoarthritis and atherosclerosis: a systematic review and meta-analysis. Exp. Gerontol..

[25] Orellana F., Grassi A., Hlushchuk R., Wahl P., Nuss K.M., Neels A. (2024 14:1. 2024). Revealing the complexity of meniscus microvasculature through 3D visualization and analysis. Sci. Rep..

[26] Villa-Bellosta R. (2018). Synthesis of extracellular pyrophosphate increases in vascular smooth muscle cells during phosphate-induced calcification. Arterioscler. Thromb. Vasc. Biol..

[27] Kosik-Bogacka D.I., Lanocha-Arendarczyk N., Kot K., Zietek P., Karaczun M., Prokopowicz A. (2018). Calcium, magnesium, zinc and lead concentrations in the structures forming knee joint in patients with osteoarthritis. J. Trace Elem. Med. Biol..

[28] Habata T., Ohgushi H., Takakura Y., Tohno Y., Moriwake Y., Minami T. (2001). Relationship between meniscal degeneration and element contents. Biol. Trace Elem. Res..

[29] Bernabei I., Sayous Y., Raja A.Y., Amma M.R., Viry A., Steinmetz S. (2021). Multi-energy photon-counting computed tomography versus other clinical imaging techniques for the identification of articular calcium crystal deposition. Rheumatology.

[30] Budzik J.F., Marzin C., Legrand J., Norberciak L., Becce F., Pascart T. (2021). Can dual-energy computed tomography be used to identify early calcium crystal deposition in the knees of patients with calcium pyrophosphate deposition?. Arthritis Rheumatol..

[31] Jarraya M., Bitoun O., Wu D., Balza R., Guermazi A., Collins J. (2024). Dual energy computed tomography cannot effectively differentiate between calcium pyrophosphate and basic calcium phosphate diseases in the clinical setting. Osteoarthr Cartil Open.

[32] Schumacher H.R. (1996). Crystal-induced arthritis: an overview. Am. J. Med..

[33] McAllister M.J., Chemaly M., Eakin A.J., Gibson D.S., McGilligan V.E. (2018). NLRP3 as a potentially novel biomarker for the management of osteoarthritis. Osteoarthr. Cartil..

[34] Busso N., So A. (2012). Microcrystals as DAMPs and their role in joint inflammation. Rheumatology.

[35] Meyer F., Dittmann A., Kornak U., Herbster M., Pap T., Lohmann C.H. (2021). Chondrocytes from osteoarthritic and chondrocalcinosis cartilage represent different phenotypes. Front. Cell Dev. Biol..

[36] Bertrand J., Kräft T., Gronau T., Sherwood J., Rutsch F., Lioté F. (2020). BCP crystals promote chondrocyte hypertrophic differentiation in OA cartilage by sequestering Wnt3a. Ann. Rheum. Dis..

[37] Mackie E.J., Ahmed Y.A., Tatarczuch L., Chen K.S., Mirams M. (2008). Endochondral ossification: how cartilage is converted into bone in the developing skeleton. Int. J. Biochem. Cell Biol..

[38] Aghajanian P., Mohan S. (2018). The art of building bone: emerging role of chondrocyte-to-osteoblast transdifferentiation in endochondral ossification. Bone Res..

[39] Kourí J.B., Aguilera J.M., Reyes J., Lozoya K.A., González S. (2000). Apoptotic chondrocytes from osteoarthrotic human articular cartilage and abnormal calcification of subchondral bone. J. Rheumatol..

[40] Chen W.H., Lo W.C., Hsu W.C., Wei H.J., Liu H.Y., Lee C.H. (2014). Synergistic anabolic actions of hyaluronic acid and platelet-rich plasma on cartilage regeneration in osteoarthritis therapy. Biomaterials.

[41] fei Yan J., pin Qin W., cheng Xiao B., qian Wan Q., Tay F.R., Niu L na (2020). Pathological calcification in osteoarthritis: an outcome or a disease initiator?. Biol. Rev..

[42] Kattimani V.S., Kondaka S., Lingamaneni K.P. (2016). Hydroxyapatite–-past, present, and future in bone regeneration. Bone Tissue Regen. Insights.

[43] Nguyen C., Bazin D., Daudon M., Chatron-Colliet A., Hannouche D., Bianchi A. (2013). Revisiting spatial distribution and biochemical composition of calcium-containing crystals in human osteoarthritic articular cartilage. Arthritis Res. Ther..

[44] Johnson K., Terkeltaub R. (2005). Inorganic pyrophosphate (PPI) in pathologic calcification of articular cartilage. Front. Biosci..

[45] Zaka R., Williams C.J. (2005). Genetics of chondrocalcinosis. Osteoarthr. Cartil..

[46] Shen G., Shen G. (2005). The role of type X collagen in facilitating and regulating endochondral ossification of articular cartilage. Orthod. Craniofac. Res..

[47] Eerola Iiro, Salminen Heli, Lammi Pirkko, Lammi Mikko, von der Mark Klaus, Vuorio Eero (1998). Type X collagen, a natural component of mouse articular cartilage: association with growth. aging, and osteoarthritis.

[48] Hubert J., Beil F.T., Rolvien T., Butscheidt S., Hischke S., Püschel K. (2020). Cartilage calcification is associated with histological degeneration of the knee joint: a highly prevalent, age-independent systemic process. Osteoarthr. Cartil..

[49] Mitsuyama H., Healey R.M., Terkeltaub R.A., Coutts R.D., Amiel D. (2007). Calcification of human articular knee cartilage is primarily an effect of aging rather than osteoarthritis. Osteoarthr. Cartil..

[50] Derfus B.A., Kurian J.B., Butler J.J., Daft L.J., Carrera G.F., Ryan L.M. (2002). The high prevalence of pathologic calcium crystals in pre-operative knees. J. Rheumatol..

