# Supplementary material for: Characterization of calcifications in posterior horn of human meniscus using micro-computed tomography
Source: Osteoarthr Cartil Open. 2026 May 16;8(3):100820. doi: 10.1016/j.ocarto.2026.100820 (PMC13234461; doi:10.1016/j.ocarto.2026.100820)
Supplement: Multimedia component 1 [file mmc1.docx]

Supplementary Material 1

1. Details on tissue samples

The samples used in this study were obtained from the knee tissue biobank MENIX, located at Skåne University Hospital in Lund, Sweden. This biobank contains meniscus samples from two distinct groups: individuals with knee OA who underwent a total knee replacement (TKR) at Trelleborg Hospital and deceased adult donors (samples obtained within 48 hours post-mortem) with no known history of knee OA. All samples in the biobank were preserved at -80 ºC within two hours of extraction.

The sample set of this study consisted of 80 meniscus samples: we selected both the medial and lateral menisci from a single knee from 19 individuals with end-stage medial compartment knee OA and 21 deceased adult donors without a known diagnosis of knee OA or rheumatoid arthritis as assessed from medical records and information provided by next of kin. Additional exclusion criteria included known drug addiction, hepatitis, or HIV infection. The donors were not screened for any other specific conditions. In the TKR group, the surgeon's Outerbridge classification of knee joint cartilage was used to assess the primary compartment affected by OA (OUTERBRIDGE, 1961). Individuals who underwent TKR with a medial grade of IV and a lateral grade lower than IV were included in the study. Additionally, the samples required a surgeon's sketch revealing at least a part of the posterior horn of the medial meniscus to be remaining, to be considered eligible for the study. Macroscopic intactness of the deceased donor menisci was also required for inclusion in the study. Given that all available TKR recipients were older than 50 years, we applied the same age threshold for deceased donors.

2. Sample preparation

The menisci were divided into two parts with a scalpel. The posterior horn with some part of the body was used in this study. The posterior horns were then fixed in 4% saline-buffered formaldehyde until they were dissected into several 5–10 mm thick pieces. The first piece was subsequently immersed in formalin, water, absolute alcohol, and xylene, then infiltrated with molten paraffin. No decalcification process was performed on the samples to preserve the calcifications. The processed samples were then manually embedded into paraffin blocks. Vertical and horizontal 4-µm-thick sections stained with Safranin O – Fast green, and hematoxylin and eosin (H&E) were used in the histopathological scoring of meniscal degeneration. In addition, vertical Alizarin red stained sections were used for visual inspection of meniscal calcifications.

3. Raman spectroscopy measurements

Raman spectroscopy measurements were done on 5-µm-thick vertical sections that were positioned on highly polished stainless steel Raman windows. Before the Raman spectral measurements, the paraffin was chemically dewaxed from each sample. The Raman spectra were acquired using the DXR 2xi Raman imaging system (Thermo Scientific, Wisconsin, USA). For measurements, 10×/0.25NA air objective and a high-resolution grating were employed, providing a spectral range of 50–1800 cm^−1^ and a spectral resolution of 5 cm^−1^. Raman scattering was initiated using a 785 nm laser set at 25 mW, directed through a 50 µm confocal pinhole aperture. Each Raman spectrum was collected for 0.25 s and averaged over 8 scans to enhance signal quality.

4. Micro-computed tomography imaging

The fixed µCT samples were first dehydrated in ascending ethanol concentrations (30%-50%-70%-80%-90%-96%-100%), treated with hexamethyldisilazane (HMDS) for 4 hours, and air-dried in a fume hood overnight. The image acquisition was performed using a desktop µCT device (SkyScan 1272, Bruker microCT, Kontich, Belgium) with the following settings: tube voltage 60kV; tube current 166µA; no additional filtering; isotropic voxel size 2.0µm; number of projections 2400; averaging 2 frames/projection; random movement 25 pixels; and exposure time 3500ms. NRecon software (Version 2.2.2.0 Bruker microCT) was used for image reconstruction. Two image reconstructions were performed for each sample with optimized settings and windowing for soft tissue and calcifications, producing two image stacks as an output. During image reconstructions, beam-hardening and ring-artefact corrections were applied together with pixel masking for defect pixels.

5. Statistical analysis

SPSS (IBM SPSS Statistics, v. 29.0, NY, USA) was used for statistical analysis. For all parameters, we did a group-wise comparison between BCP and CPP groups using linear mixed models. All parameters were transformed into logarithms before calculating arithmetic mean for each meniscus which was used as outcome in the statistical models. The results were back transformed and thus, the difference between groups are expressed as ratios of geometric means. Used parameters were set as dependent variable and we used a random effect to account for multiple menisci from the same knee (menisci nested within persons). For total calcification volume and total number of particles, we adjusted for the meniscus logarithm tissue volume to account for differing sizes of meniscus. For closed porosity, volume of closed porosity was set as dependent variable, and the logarithm of total calcification volume was used as a fixed variable to account for differing total volumes of calcification. We did not adjust for any other covariates due to low number of menisci with CPP calcifications. We checked the assumptions of the linear mixed models using plots of residuals, QQ plots for normality of residuals and random effects and residuals vs fixed fitted values plot to assess linearity and homoscedascticity. We found no evidence of violation of the assumptions.


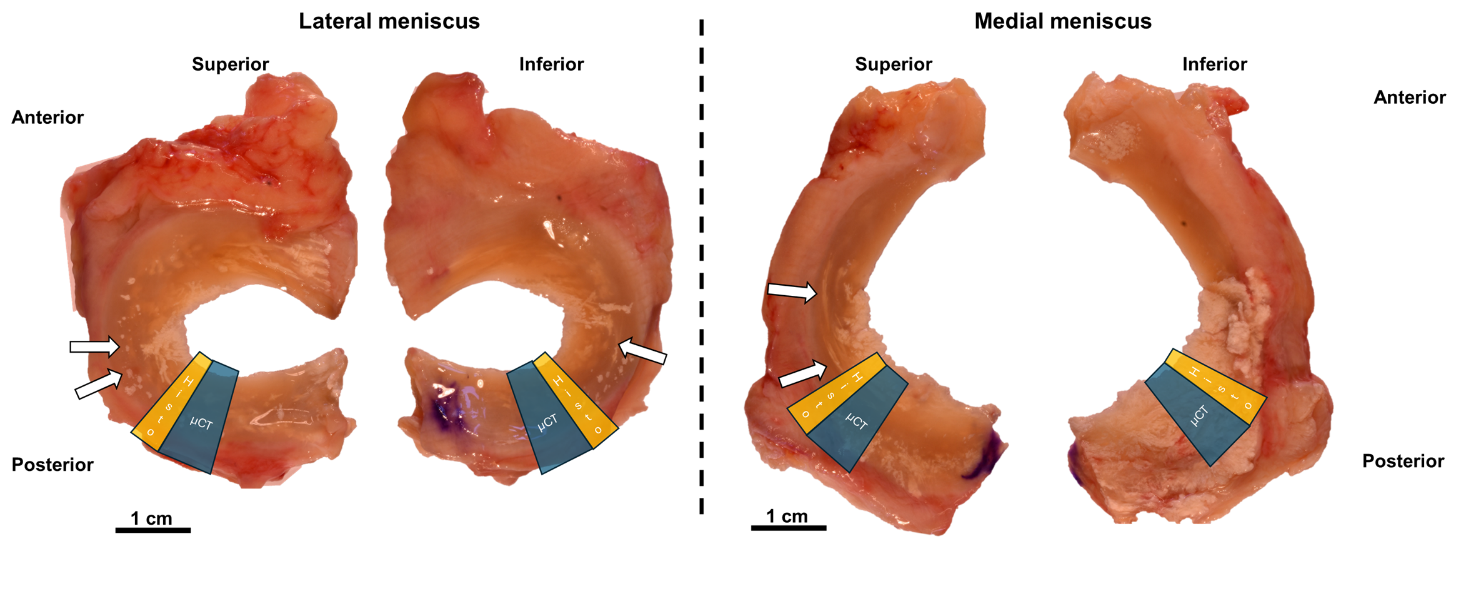


Supplementary Figure S1. An example of deceased donor’s lateral and medial meniscus. Rod-like calcium pyrophosphate (CPP) calcifications can be seen inside the tissue (arrows), following the circumferential shape of meniscus. Location of the micro-computed tomography (µCT) piece is shown in blue. Location of cut sections for histological analysis and Raman spectroscopy is shown in yellow.


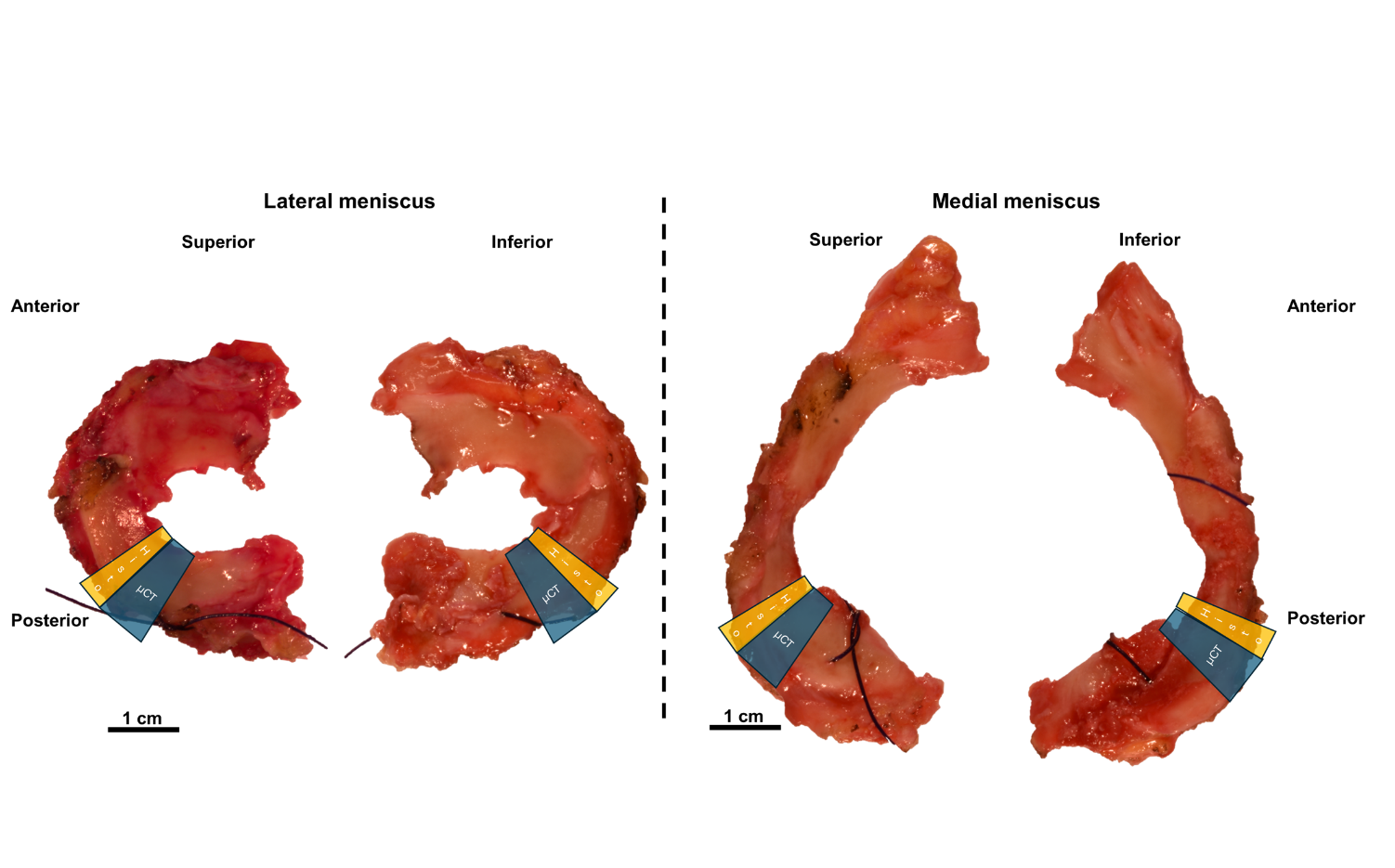


Supplementary Figure S2. An example of a lateral and medial meniscus from a total knee replacement patient with medial compartment osteoarthritis with Outerbridge score one on lateral side and four on medial side. No visible calcifications are seen. Location of the micro-computed tomography piece is shown in blue. Location of histological sections and Raman spectroscopy is shown in yellow.


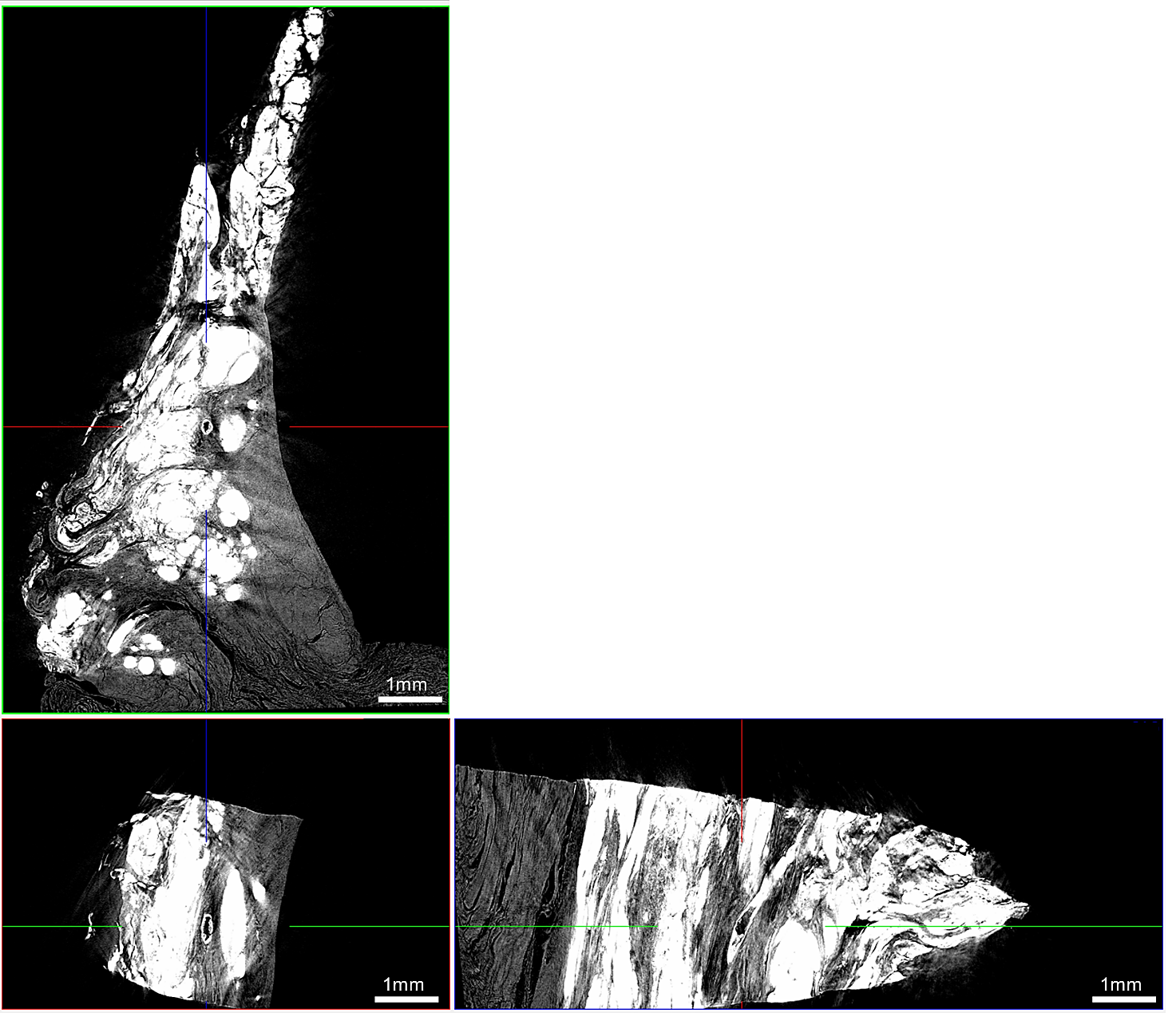


Supplementary Figure S3. An example cross-section from three directions of a hollow rod-shaped calcification in a sample with identified CPP calcifications from a donor meniscus with a Pauli score of 14.

6. Description of calcifications, cells, and proteoglycan content,

An example meniscus sample with both BCP (blue arrow) and CPP (red arrow) calcifications identified with Raman spectroscopy is shown in supplementary figure S4. The BCP calcifications in general were found attached to the surface, fibrillations, or periphery of the meniscus. Figure S4B shows distinct shape and density differences between BCP and CPP aggregates, with BCP appearing denser and CPP accumulating in rod-like shapes, covering the whole sample from the anterior to the posterior side, while BCP forms clusters with sharp edges. In Figure S4C, adjacent histology sections shows BCP (blue arrow) only as a small cluster in the periphery of the meniscus outer region. CPP (red arrow and all neighboring calcifications) are seen inside the meniscus and near teared area. In Figure S4E, the left arrow shows amorphous CPP calcification, while the right arrow shows solid, circumferential rod-like CPP. In Figure S4G, BCP is located in the periphery of the outer region of meniscus, with high cell count in the joint capsule with vascular supply and only a few cells in the outer region. In Figure S4H, the cells around solid CPP are ellipsoid-shaped and oriented along the calcification surface, similar to chondrocytes in articular cartilage. Importantly: there are no distinct cells or lacunae structures or staining visible inside the BCP or CPP calcifications in histological sections (Supplementary figure S4 H&K). Safranin O staining intensity is increased in the whole sample (Supplementary figure S4I). However, the areas near solid CPPs are more pronounced in staining, compared to more amorphous CPP areas (Supplementary figure S4 J&K).
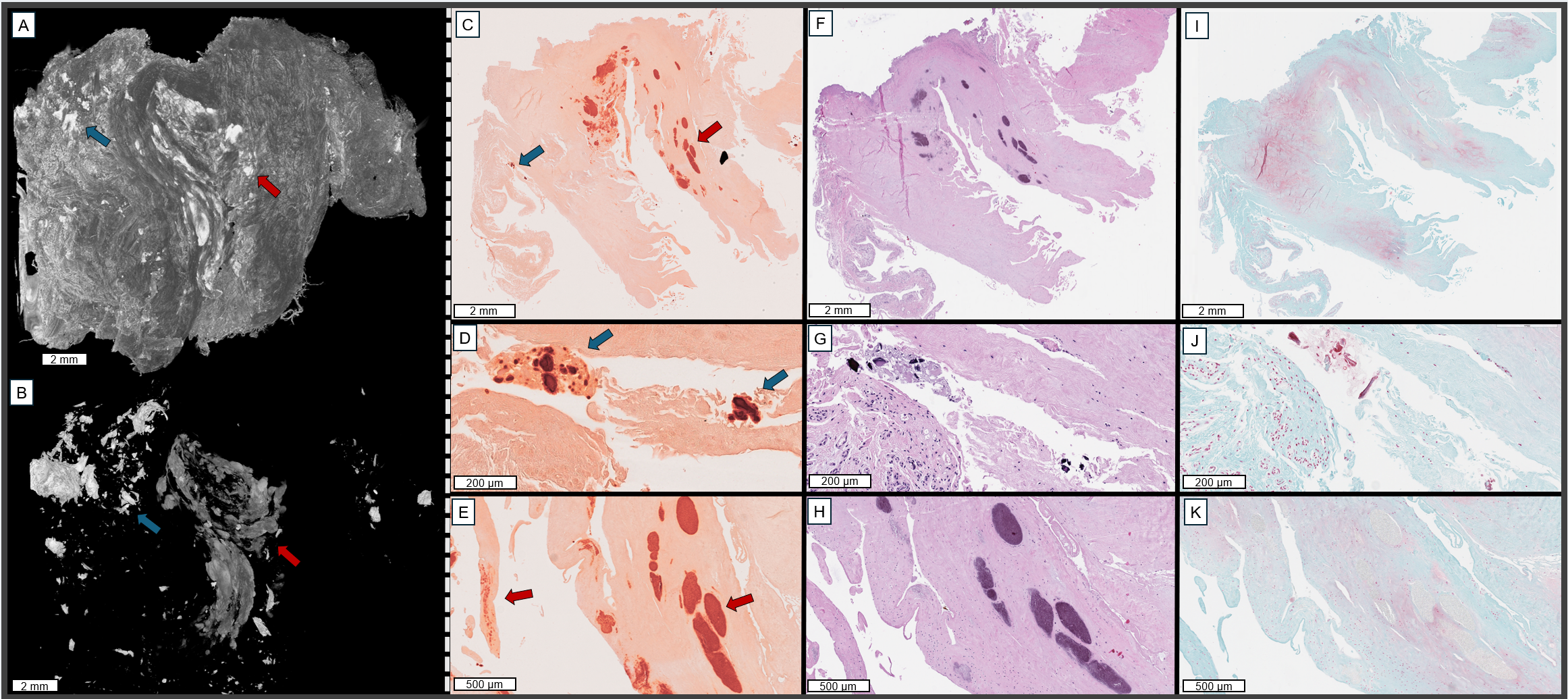
Supplementary figure S4. A) A representative 3D µCT image of meniscus from individual with OA, with a Pauli score of 16, with both BCP (blue arrow) and CPP (red arrow) calcifications, as identified with Raman spectroscopy measurements of an adjacent tissue section. B) 3D µCT image of the calcifications without soft tissue. C-E) Alizarin Red -stained histological section adjacent to the µCT piece. C) BCP highlighted with blue arrow and CPP with red arrow. D) Magnified image of BCP from 3C in slightly different orientation. E) Magnified image from 3C of CPP with amorphous CPP on the left and solid, ellipsoidal CPP on the right. F, G, H) Adjacent hematoxylin and eosin -stained sections show cell nuclei near calcifications. I, J, K) Adjacent Safranin O - Fast Green -stained sections show increased proteoglycan staining in the whole tissue, while solid CPP areas have more Safranin O staining around them compared to the surroundings of amorphous CPP areas.

Supplementary figure S5 A&B shows that the particles are located in the complex surface fibrillations and tears of the meniscal surface of a meniscus sample from an individual with OA inhabiting only BCP calcification. From histological sections in Figure S4C-E, the complex 3D tears are not visible and the BCP particles are seemingly inside the sample. In Figure S4F-H, the cells seem hypertrophic, and only a few normal, healthy cells are seen around the BCP aggregates. Safranin O staining in Figures S4I-K reveals increased staining in the whole sample. Similar to the sample in Figure S4, the BCP calcifications do not show distinct porosity inside their structure in histological sections.


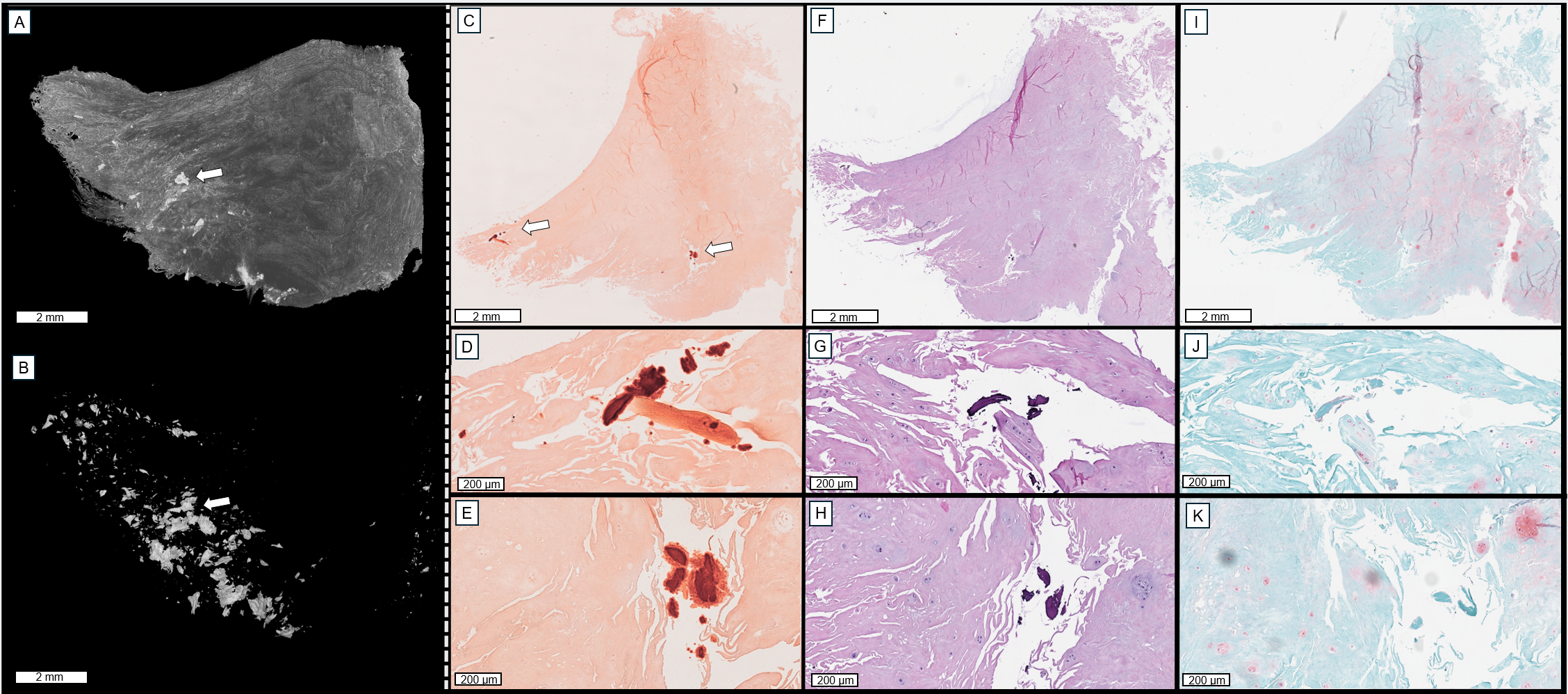


Supplementary figure S5. A) A representative 3D µCT image of a meniscus sample with a Pauli score of 16, from an individual with OA inhabiting only BCP calcifications. B) The calcifications are inside the fibrillations and tears near the meniscus surface, and a few punctate calcifications are seen on the outer region. C, D, E) Alizarin red staining shows two distinct calcification clusters that are seemingly inside the meniscus. F, G, H) Hematoxylin and eosin staining shows few hypertrophic cells and loss of healthy cells around the tear, where BCP calcifications are located. I, J, K) Safranin O - Fast Green staining shows an increase in proteoglycan content and oedema-like features in the sample.


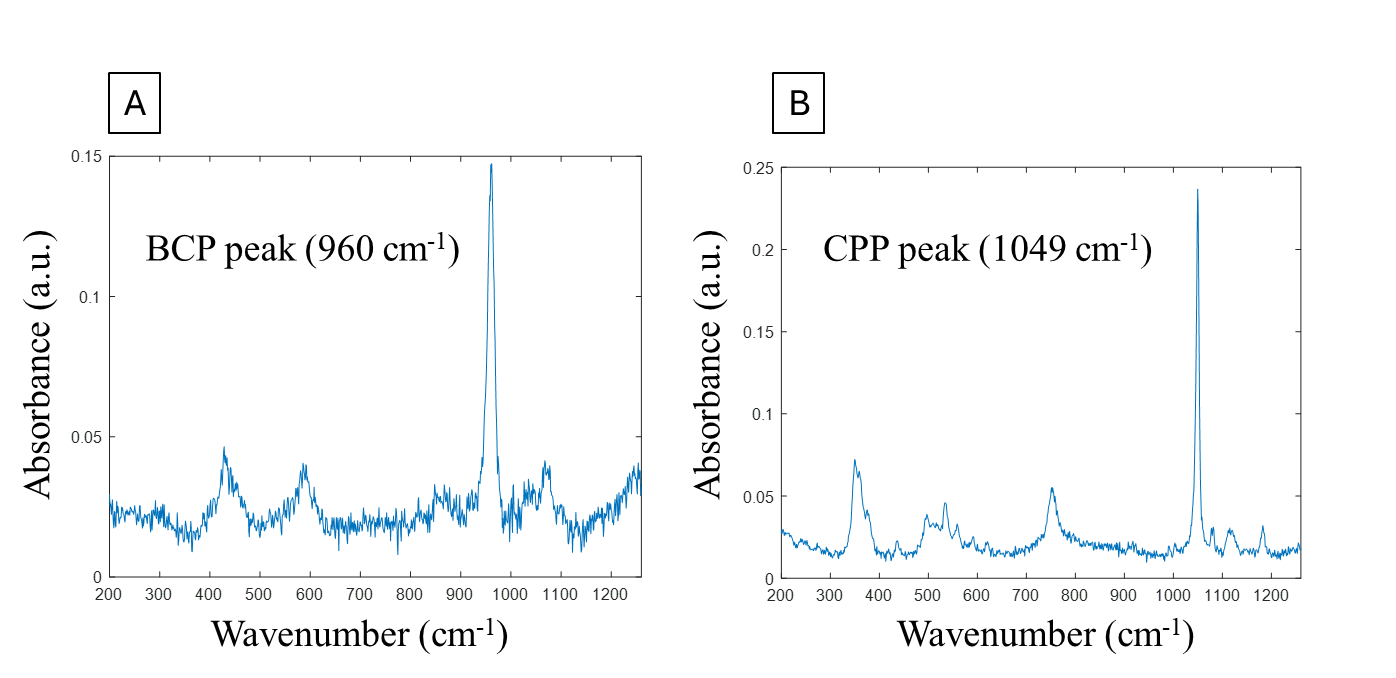


Supplementary Figure S6. A) Example of BCP spectra from Raman microspectroscopy measurement from BCP area in supplementary figure S4. B) Example of CPP spectra from CPP measurement from CPP area in supplementary figure S4.

7. Inter-observer reliability

The inter-observer reliability of calcification grading was assessed between the three readers using Cohen's linearly weighted kappa coefficients (κw) and their 95% confidence intervals (CIs). Lateral side for graders 1 and 2: κw = 0.73 (95% CI 0.61, 0.86), for graders 1 and 3: κw = 0.70 (95% CI 0.57, 0.84), and for graders 2 and 3: κw = 0.91 (95% CI 0.84, 0.98). Medial side for graders 1 and 2: κw = 0.75 (95% CI 0.65, 0.84), for graders 1 and 3: κw = 0.73 (95% CI 0.64, 0.82), and for graders 2 and 3: κw = 0.93 (95% CI 0.87, 0.98).


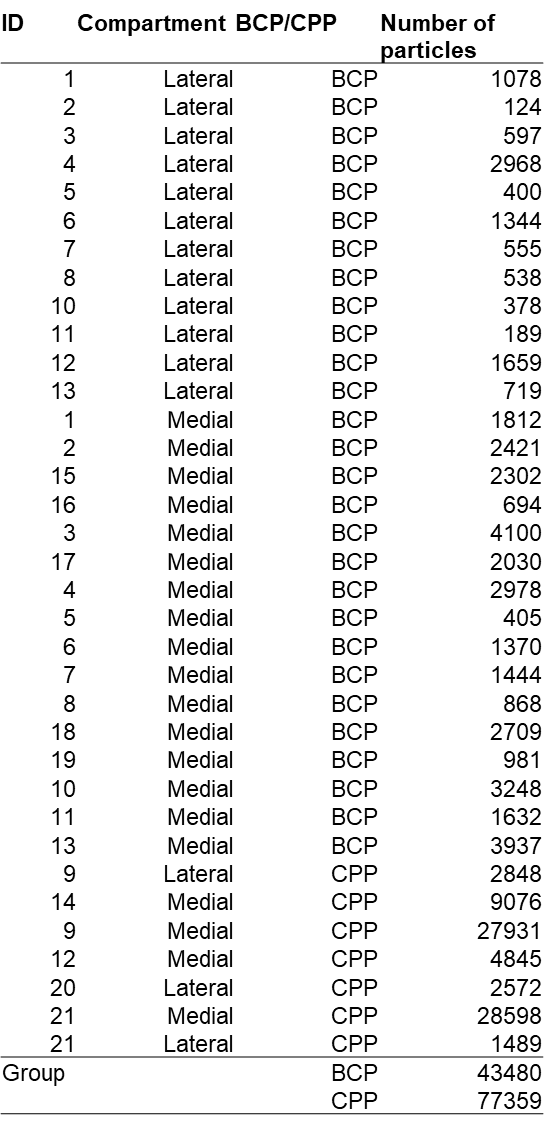


Supplementary Figure S7. Total number of analyzed particles for each meniscus.

References

OUTERBRIDGE, R. E. (1961). The etiology of chondromalacia patellae. *The Journal of Bone and Joint Surgery. British Volume*, *43-B*(4), 752–757. https://doi.org/10.1302/0301-620X.43B4.752/LETTERTOEDITOR
