# Supplementary material for: Characterization of calcifications in posterior horn of human meniscus using micro-computed tomography
Source: Osteoarthr Cartil Open. 2026 May 16;8(3):100820. doi: 10.1016/j.ocarto.2026.100820 (PMC13234461; doi:10.1016/j.ocarto.2026.100820)
Supplement: Multimedia component 2 [file mmc2.docx]

**Video legends**

Supplementary Video 1: Basic calcium phosphate (BCP) calcifications in an OA meniscus with a Pauli score of 16 are mainly located on the surface and inside fibrillations. They appear mostly aggregated as punctuate and small in size (5-100µm in diameter), while larger clusters can be over 500 µm in largest diameter and have characteristically sharp and pointy edges.

Supplementary Video 2: Calcium pyrophosphate (CPP) covers most of the donor meniscal tissue (Pauli score 13). Cross-sectional diameter of solid rod-like CPP calcifications was commonly between 0.2-1mm. Largest calcifications accumulate between the circumferential collagen fiber bundles, aligning the circumferential fibers, while forcing the collagen to warp around them.

Supplementary Video 3: Calcium pyrophosphate (CPP) covers most of the donor meniscal tissue (Pauli score 14) forming long rod-like calcifications. Moreover, few cases of long and hollow calcifications are observed inside the meniscus. Additionally, CPP calcifications have generally smooth surfaces, but amorphous, less dense structures of CPP are seen near the rod-shaped calcifications and on the surface of the meniscus.

Supplementary Video 4: Close-up video of basic calcium phosphate (BCP) calcifications and pore structures in OA meniscus with Pauli score 16. The closed pores are highlighted in red, showing how the pore organization is constant.
